# Quantification of recombinant immunotoxin delivery to solid tumors allows for direct comparison of *in vivo* and *in vitro* results

**DOI:** 10.1038/srep10832

**Published:** 2015-06-26

**Authors:** Emily Mason-Osann, Kevin Hollevoet, Gerhard Niederfellner, Ira Pastan

**Affiliations:** 1 Laboratory of Molecular Biology, Center for Cancer Research, National Cancer Institute, National Institutes of Health, Bethesda, Maryland, USA; 2 Roche Pharmaceutical Research & Early Development, Discovery Oncology, Innovation Center Penzberg, Roche Diagnostics GmbH, Nonnenwald 2, 82377 Penzberg, Germany

## Abstract

Solid tumors present challenges for delivery of protein therapeutics; current methods cannot quantify the functional effects of these agents. RG7787 (anti-mesothelin recombinant immunotoxin) is highly cytotoxic to pancreatic cancer cell lines, but with limited activity *in vivo*. To investigate this discrepancy, we developed a flow cytometry method to quantify the amount of RG7787 internalized per cell in tumors and used it to analyze tumor responses by determining the number of molecules of RG7787 internalized per cell *in vivo* and comparing it to that needed to kill cells *in vitro*. At a maximum tolerated dose of 7.5 mg/kg, tumor cells *in vivo* internalized a wide range of RG7787 with the average amount equivalent to the amount that induced growth arrest *in vitro*. However, 20% of cells accumulated 20,300 ITs per cell, sufficient to kill cells *in vitro*. At 2.5 mg/kg the top 20% of cells internalized enough RG7787 to only induce growth arrest. These data are in agreement with tumor responses; 22% regression following a 7.5 mg/kg dose and growth stabilization following 2.5 mg/kg. Comparing amounts of RIT delivered *in vivo* and *in vitro* can explain tumor responses and should facilitate the development of more active immunotoxins and other antibody based agents.

Recombinant immunotoxins (RITs) are a class of biological agents being developed for the treatment of cancer. They are chimeric proteins consisting of a cytotoxic portion of *Pseudomonas* exotoxin A fused with an Fv or Fab that targets antigens on cancer cells^1^. RITs rely on cellular internalization for activity, unlike immunotherapy with unarmed whole antibodies^2^.

It is well known that tumor penetration of antibodies and antibody conjugates are inhibited by the physical and biological properties of solid tumors^3^,^4^,^5^. These include the lack of functional lymphatics, high interstitial pressure, irregular vascularization^4^,^5^,^6^,^7^, and a binding site barrier^8^,^9^. Current methods for measuring drug delivery are lacking in sensitivity, resolution, or quantification. Administration of radiolabeled antibodies can quantify changes in penetration into tumors and assess biodistribution^9^,^10^,^11^, but does not measure drug delivery to individual cancer cells. Fluorescence based methods, such as confocal microscopy and immunofluorescence allow direct visualization at the cellular level and are useful for analysis of spatial distribution of therapeutics in tissue, but only quantify relative amounts of accumulation^5^,^12^.

We have been studying a RIT named SS1P that targets mesothelin, a cell surface glycoprotein highly expressed on many malignancies, including mesothelioma, ovarian cancer, triple negative breast cancer and pancreatic cancer^13^. While SS1P had very modest anti-tumor effect as a single agent in clinical trials, it produced striking responses in a subset of patients when combined with immune-suppressive therapy, which prevented anti-drug antibody formation, and allowed more doses to be given^14^. To decrease immunogenicity and side effects that limit SS1P therapy^15^,^16^, we have developed a clinically-optimized anti-mesothelin RIT (RG7787) in collaboration with Roche Pharma Research and Early Development (Fig. 1A)^13^,^17^,^18^. RG7787 is highly active *in vitro* against several pancreatic ductal adenocarcinoma (PDAC) cell lines, including KLM-1. When tested on KLM-1 tumors in mice, RG7787 produced minor tumor regressions as a single agent, and profound tumor regressions when combined with paclitaxel^13^. One possible explanation for RG7787’s failure to produce profound regressions as a single agent is that insufficient concentrations of RIT reach tumor cells. Because current methods are insufficient for quantifying amounts of RIT or other antibody based agents that are delivered *in vivo* to tumor cells, we have developed a method to do this and applied it to a pancreatic cancer.

Previously, we measured the percentage of cancer cells in an A431/H9 tumor that had internalized fluorescently labeled SS1P, by enzymatically digesting tumors from treated mice^19^. We used a labeled antibody against human EGFR to discriminate tumor cells from murine cells (like macrophages) that non-specifically internalize immunotoxin^19^. Using an untreated tumor to establish a threshold to distinguish cells that had internalized SS1P from those which had not, we measured the percentage of tumor cells positive for immunotoxin. This method is dependent on very high amounts of EGFR on the cell surface, which does not occur in most cancer cells. Also because the fluor used to label SSIP is not very bright, we could not detect cells that had taken up small amounts of RIT.

We have now developed an improved method that allows one to calculate the number of molecules internalized by single KLM-1 tumor cells, which enables us to explain why RG7787, which is very toxic to KLM1 cells *in vitro*, causes only a modest decrease in the size of KLM1 tumors^13^.

## Results

### Quantifying RG7787-Alexa Fluor 647 internalization in xenografts

We have shown that RG7787 alone is highly cytotoxic to KLM-1 cells *in vitro*, but produced minor but not major tumor regressions *in vivo*^13^. To explain this discrepancy, we hypothesized that insufficient amounts of immunotoxin were reaching the tumor cells *in vivo*. To test this hypothesis, we needed to develop a general method to quantify the amount of RIT delivered to individual cancer cells in solid tumors growing in mice and then compare this data with *in vitro* data where total cell killing can be achieved.

We have carried out experiments to measure immunotoxin uptake by tumors, but were only able to determine the percentage of cancer cells containing fluorescently labeled RIT. Furthermore to identify the cancer cells, we used an antibody to EGFR^13^,^19^. This approach is only useful for cells expressing very high levels of EGFR, which is very uncommon. To make the method generally useful we made 3 changes. We used CD71 (human transferrin receptor) to identify tumor cells, we used Sytox blue to identify and exclude dead cells, and we replaced Alexa Fluor 488 with the much brighter Alexa Fluor 647. A brief description of these modifications was included in a recent publication^13^. The current study reports complete details of the method and more importantly its use to relate tumor responses to *in vitro* activity of immunotoxin RG7787.

**Figure 1:**
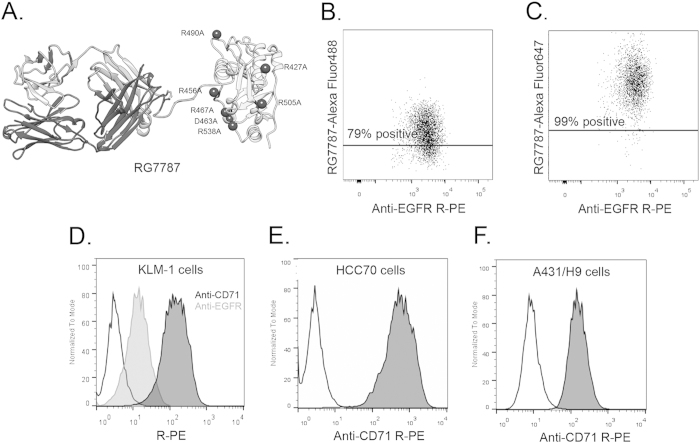
Optimization of flow cytometry method for measuring RIT internalization. **A**. Structural model of anti-mesothelin immunotoxin RG7787. Model is hypothetical and previously described in detail^13^. RG7787 contains a humanized SS1 Fab, shown on the left, linked to a small portion of domain II (processing) and all of domain III (catalytic) of Pseudomonas Exotoxin A through a GGS linker containing a furin cleavage site. Domain III contains 7-point mutations to silence B-cell epitopes. B-C, A431/H9 tumor cell population (anti-EGFR R-PE+) 3 hrs after treatment with 5.85 mg/kg RG7787-Alexa Fluor 488 (**B**) or RG7787-Alexa Fluor 647 (**C**). Line is drawn above level at which untreated control tumor cell population has 2-5% positivity (data not shown). **D**. Histogram showing unstained KLM-1 cells (black, unfilled), stained with anti-EGFR R-PE (gray line, filled), or anti-CD71 R-PE (black line, filled). **E**. Triple negative breast cancer, HCC70 cells stained with anti-CD71 R-PE. **F.** Mesothelin transfected epidermoid cancer cell, A431/H9 cells stained with anti-CD71 R-PE.

Figure 1B,C show the tumor cell population of A431/H9 tumors in mice treated for 3 hours with 5.85 mg/kg i.v. RG7787-Alexa Fluor 488 or RG7787-Alexa Fluor 647. Following treatment with Alexa Fluor 488-RG7787, 79% of the tumor cells fall above a threshold established based on an untreated tumor (Fig. 1B) compared to 99% for Alexa Fluor 647-labeled RG7787 (Fig. 1C). This indicates that measuring percentage of cells positive for immunotoxin is dependent on brightness of the fluor, and this improvement allows detection of cells containing small amount of immunotoxin.

Figure 1D shows that KLM-1 cells identified by staining for EGFR showed only a 3.9-fold signal increase over background, indicating low expression levels of surface EGFR. As a consequence stained and unstained cells cannot be clearly distinguished *in vitro* or in KLM-1 tumors (Supporting Information Fig. 1A). To overcome this limitation, we have utilized the transferrin receptor, which is ubiquitously and highly expressed on the surface of many types of cancers^20^,^21^,^22^,^23^,^24^,^25^,^26^. When we stained for CD71 instead of EGFR to identify KLM-1 tumor cells, we found that KLM-1 cells stained with anti-CD71 R-Phycoerythrin (R-PE) were 40-fold brighter than background and have a peak clearly distinguishable from unstained cells, unlike the cells stained for EGFR (Fig. 1D). Additionally, we assessed the use of transferrin receptor to identify other cancer cell types. Triple negative breast cancer cells, HCC70, stained with anti-CD71 R-PE had a 170-fold increase (Fig. 1E) and mesothelin-transfected epidermoid carcinoma cells, A431/H9, had a 17-fold increase over background signal (Fig. 1F). While A431/H9 had a lower signal than the other cell lines, the stained and unstained peaks were still distinct with limited overlap. This indicates that a 17-fold increase in signal over background is sufficient to identify CD71 stained cells, and therefore tumor cells. Enzyme-digested HCC70 or A431/H9 tumors stained with anti-CD71 R-PE can be seen in Supporting Information Fig. 1. In summary, these data show that CD71 staining can identify human cancer cells from unstained murine stromal cells in several different xenograft models.

### Quantification of number of RG7787 molecules internalized in KLM-1 tumors

**Figure 2:**
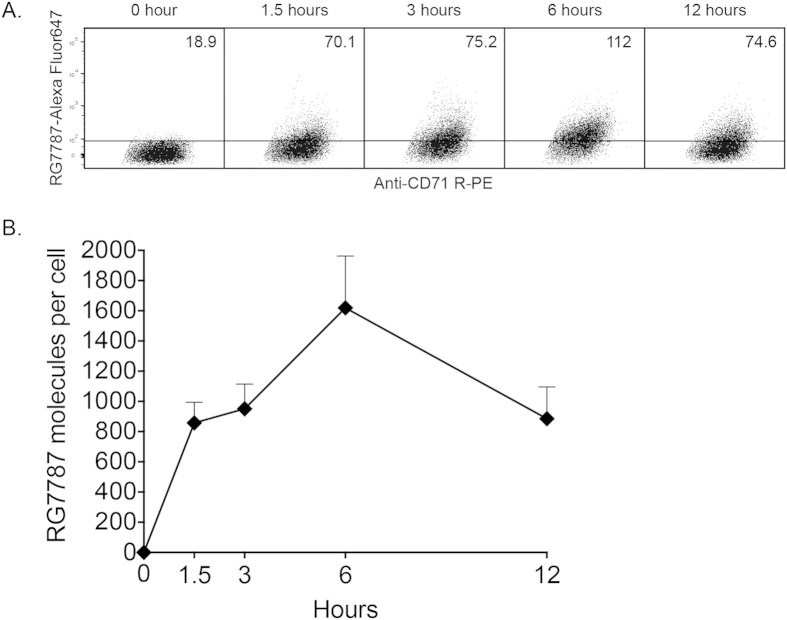
Quantification of RG7787 molecules per tumor cell. **A**. Representative dot plots of KLM-1 xenografts (120-140 mm^3^) excised after treatment with 2.5 mg/kg i.v. RG7787-Alexa Fluor 647. Line is drawn above untreated tumor. The number in the top right corner of each frame is the MFI of Alexa Fluor 647 for KLM-1 (Sytox negative, CD71 R-PE positive). **B**. MFI values translated into average RG7787-Alexa Fluor 647 molecules per cell at each time point.

Previously, we measured tumor penetration by quantifying the percentage of KLM-1 cells internalizing RG7787^13^. In the current study, we measured the amount of RIT internalized per tumor cell, which allowed us to understand the functional effects of internalized RIT *in vivo*. We used Alexa Fluor 647 Molecules of Equivalent Soluble Fluorochrome beads (MESF; Bangs Laboratories) to establish a standard curve, and interpolated the geometric mean fluorescence intensity (MFI) of KLM-1 tumor cell populations on this curve using Bangs Laboratories QuickCal software (Supporting Information Fig. 2) to calculate RG7787 molecules per cell. While the previously reported method estimated the number of internalized molecules based on saturation staining^19^, calibrated beads increase accuracy over a wider range of values by generating a standard curve. We subtracted the MESF value of an untreated tumor to correct for the auto-fluorescence of cells. Figure 2A shows representative dot plots of the tumor cell population after treatment with 2.5 mg/kg i.v. RG7787-Alexa Fluor 647. The MFI of the tumor cell population increased over time, indicating increased amounts of RIT internalized. MFI was converted to molecules per cell in Fig. 2B, showing that KLM-1 tumor cells internalized an average of 1,620 ± 340 RG7787 molecules per cell 6 hours after treatment. This is the time when maximal internalization occurs in this tumor^13^.

### Dose responsiveness of internalization quantification

**Figure 3:**
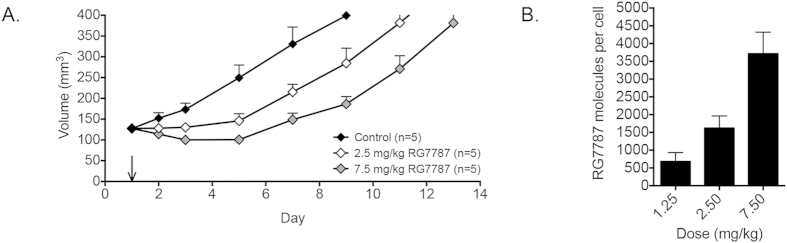
Anti-tumor activity and corresponding quantification of RG7787 molecules per cell. **A**. Tumor volumes of mice with KLM-1 xenografts (120-140 mm^3^) treated with a single dose of D-PBS 0.2% HSA (control, black), 2.5 mg/kg RG7787 (white) or 7.5 mg/kg RG7787 (gray) i.v. **B**. Average RG7787-Alexa Fluor 647 molecules per cell in KLM-1 tumor cell population (CD71 R-PE +) 6 hrs after treatment with 1.25 mg/kg (n = 3), 2.5 mg/kg (n = 7) or 7.5 mg/kg (n = 4) RG7787-Alexa Fluor 647 i.v.

The maximum dose of RG7787 that can be given safely to mice is 2.5 mg/kg every other day x3; however 7.5 mg/kg can be given safely as a single dose. We assessed the functional effect of increased RIT delivery following treatment with a higher dose by measuring the volume change of KLM-1 xenografts (Fig. 3A). A single 2.5 mg/kg dose arrested tumor growth, but did not cause tumor regression. In contrast, a single 7.5 mg/kg dose induced a 22% reduction in tumor volume measured on day 3 (100 ± 8 mm^3^ compared to 128 ± 7 mm^3^ starting volume, p = 0.0004). Starting on day 3 there was a statistically significant difference between tumor volumes from mice treated with 2.5 mg/kg and 7.5 mg/kg (131 ± 10 mm^3^ and 100 ± 8 mm^3^, respectively, p = 0.047). Treatment with a 7.5 mg/kg dose also increased the amount of internalized RG7787 to 3,710 ± 610 RG7787 molecules per cell from 1620 ± 340 when measured 6 hours following treatment (Fig. 3B), consistent with the better anti-tumor response. We then analyzed the relationship between the amount of immunotoxin internalized and the tumor response by determining how much RG7787 needs to be internalized in order to kill cells *in vitro*, and how that amount relates to the amount of RG7787 internalized in tumors in the mice.

### Characterizing *in vitro* internalization of RG7787 in KLM-1

**Figure 4:**
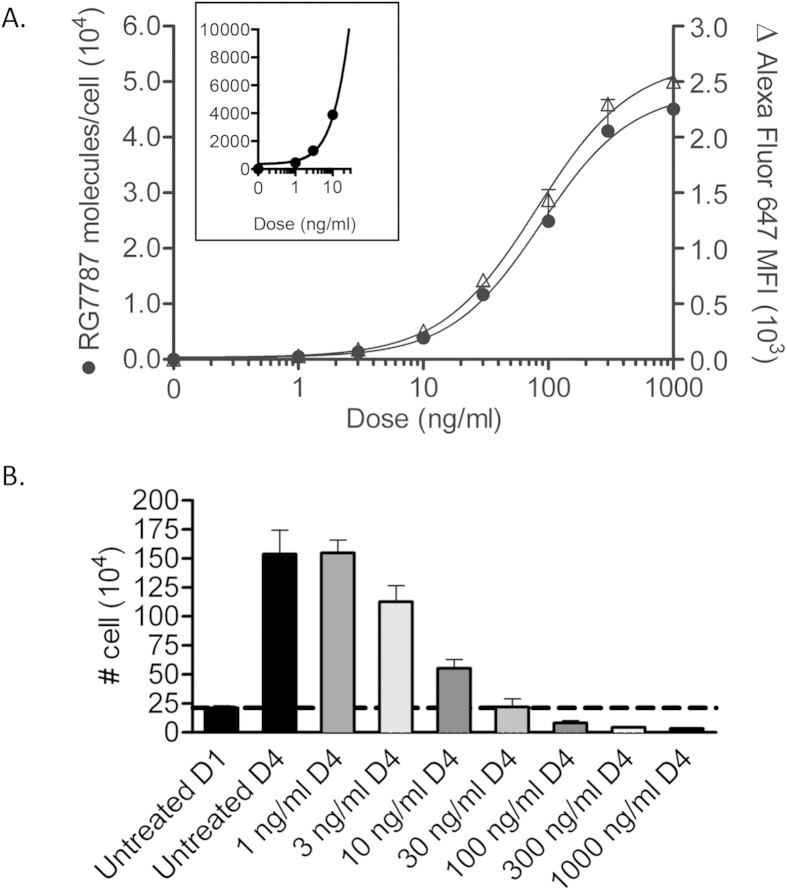
*In vitro* characterization of RG7787 internalization and cell killing in KLM-1 cells. **A**. *In vitro* internalization of RG7787-Alexa Fluor 647 in KLM-1 cells measured by flow cytometry (right axis Δ). Alexa Fluor 647 signal intensity was translated to RG7787 molecules per cell (left axis •), creating a standard curve that was used to calculate *in vitro* dose equivalents for *in vivo* treatments. **B**. Cells were counted in order to assess cell killing by RG7787. Viable cells were counted in triplicate using trypan blue to exclude dead cells. Dotted line represents the number of cells on day 1 (D1) at the time of treatment. Counts above that line on D4 represent overall growth inhibition; counts below line on D4 indicate fewer cells on D4 than D1, confirming cell death. Experiments were done in triplicate.

The amount of RG7787 internalized by KLM-1 tumor cells is dependent on the amount of RIT that enters the tumor mass, binds to the tumor cells and is internalized after binding. To determine how much immunotoxin is internalized at different doses *in vitro*, we treated KLM-1 cells with various concentrations of RG7787-Alexa Fluor 647 for 24 hours, the point at which internalization is saturating (Supporting information Fig. 3). Toxin-induced apoptosis interferes with uptake measurements at later time points. Figure 4A shows that as concentration increased, the Alexa Fluor 647 MFI also increased, indicating more RG7787 was internalized over time. Cells treated with 300 ng/ml did not have a statistically significant difference in MFI from the cells treated with 1,000 ng/ml (2,300 ± 282 and 2,500 ± 270, p = 0.63), establishing 300 ng/ml as a saturating dose. KLM-1 cells treated with 300 ng/ml for 24 hours internalized an average of 41,100 ± 5,609 molecules RG7787 per cell (Fig. 4A).

### Assessing *in vitro* activity of RG7787 on KLM-1

To assess KLM-1 cell viability after different amounts of RG7787 had been internalized (Fig. 4A), we treated KLM-1 cells with increasing amounts of RG7787 for 24 hours and counted surviving cells 72 hours later, allowing time for the internalized RIT to induce apoptosis. Fig. 4B shows average cell counts from three replicate experiments. 21 × 10^4^ untreated cells seeded on day 1 had expanded to 150 × 10^4^ viable cells on day 4. Treatment with 3 ng/ml decreased the number of viable cells by day 4 to 110 × 10^4^ showing that low doses of RG7787 cause growth inhibition. Treatment with 30 ng/ml caused complete growth inhibition with no change in the number of viable cells between days 1 and 4 (p = 0.94).

Treatment with higher doses caused cell death resulting in a decrease in viable cell number by day 4. Treatment with 100 ng/ml, 300 ng/ml or 1,000 ng/ml decreased viable counts to 8.1 × 10^4^, 4.3 × 10^4^, and 3.3 × 10^4^, respectively. Similar to *in vitro* internalization assays, the effects of 300 ng/ml and 1,000 ng/ml on cell death are similar, indicating that 300 ng/ml has maximal activity.

### Quantification of internalized immunotoxin at higher resolution

**Figure 5:**
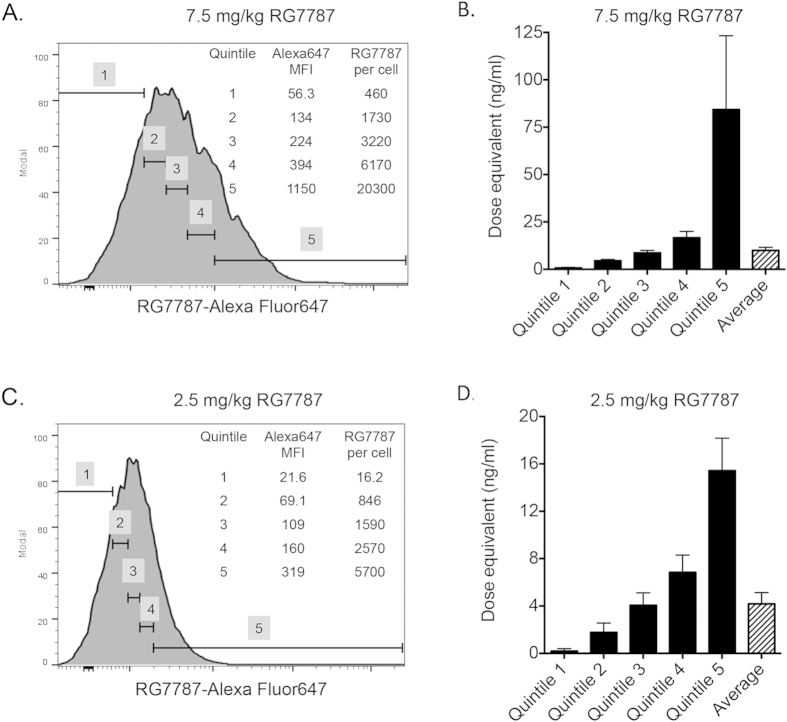
Partitioning of RIT internalization in KLM-1 tumors and relating *in vivo* RIT internalization to *in vitro* internalization to determine *in vitro* dose equivalents. Representative histogram of RG7787-Alexa Fluor 647 signal intensity of KLM-1 tumor cell population 6 hrs after i.v. treatment with 7.5 mg/kg (**A**) or 2.5 mg/kg (**C**) showing tumor cell population is divided into five equal quintiles based on MFI. MFI for each quintile is shown, and translated into average RG7787 molecules per cell for the quintile. Dose equivalents for each quintile calculated by interpolating average number of RG7787 molecules per cell for each quintile on to Fig. 4A. Dose equivalents calculated for amount of RIT internalized 6 hrs following i.v. treatment with 7.5 mg/kg (**B**) and 2.5 mg/kg (**D**). Each *in vivo* measurement is the mean of at least triplicate experiments.

The data in Fig. 2A demonstrate that there is a large range of Alexa Fluor 647 signal intensity 6 hours after treatment, indicating that some KLM-1 cells internalized much more RG7787 than others. To assess these differences, we divided the KLM-1 tumor cells into 20% quintiles according to the average amount of RG7787-Alexa Fluor 647 internalized, ranging from low (Quintile 1) to high (Quintile 5). Figure 5A shows the fluorescence intensity of each quintile of the tumor cell population 6 hours following a single 7.5 mg/kg treatment, and the corresponding number of RG7787 molecules internalized. Quintile 5 has an MFI of 1150, which is equivalent to 20,300 molecules of RG7787 per cell. Quintile 1 internalized an average of 460 immunotoxins per cell.

Figure 5C shows the amount of RG7787 taken up 6 hours after a single dose of 2.5 mg/kg RG7787 Alexa Fluor 647. Quintile 5 internalized 5,700 molecules RG7787 per cell, while quintile 1 only internalized an average of 16 molecules of RG7787 per cell. These data show that this method can quantify both the average numbers of RIT molecules delivered to a whole tumor, the amount of drug delivered to portions of a tumor population, and even to single cells.

### Comparing and relating *in vivo* and *in vitro* treatment

If we use the average amount of RG7787 internalized 6 hours after a dose of 7.5 mg/kg, the population of tumor cells internalized an average of 3,710 molecules of RG7787 per cell (Fig. 3B). KLM-1 cells internalized the same number of molecules per cell *in vitro* when treated with a dose of 10 ng/ml for 24 hours (Fig. 4A). This indicates that the tumor is exposed to the equivalent of an *in vitro* dose of 10 ng/ml, when treated intravenously with 7.5 mg/kg (Fig. 5B). Following 2.5 mg/kg *in vivo* treatment, the tumors cells are exposed to an average equivalent *in vitro* dose of 4.2 ng/ml (Fig. 5D). Both of these doses only induce cell growth arrest *in vitro*; with no decrease in the number of viable cells (Fig. 4B). However this type of analysis does not take into account the variability in uptake demonstrated in Fig. 2A.

Following treatment with 7.5 mg/kg, quintile 5 was exposed to an equivalent *in vitro* dose of 84 ng/ml (Fig. 5B), which is a dose that greatly decreased the number of viable cells *in vitro* between days 1 and 4 (Fig. 4B). This indicates that a single 7.5 mg/kg dose delivered enough RIT to at least 20% of the tumor to kill a significant number of cells. This is supported by the 22% tumor regression observed following this treatment (Fig. 3A). Lower quintiles internalized equivalents of 16.7 ng/ml, 8.6 ng/ml, 4.5 ng/ml and 0.7 ng/ml *in vitro* doses. These doses are sufficient to cause growth arrest.

Quintile 5 of the 2.5 mg/kg treatment group was exposed to an equivalent *in vitro* dose of 15.3 ng/ml (Fig. 5B), which is insufficient to induce cell death (Fig. 4B). Lower quintiles received even less. Some of these doses can induce growth arrest but not a decrease in cell number, explaining the lack of tumor regression following a single 2.5 mg/kg treatment.

Cells must receive a dose of 30 ng/ml or higher in order to decrease cell number and not just arrest overall growth, and a dose of 300 ng/ml for maximum cytotoxic effects (Fig. 4B); this translates into 11,700 and 41,100 molecules of RG7787 per cell, respectively (Fig. 4A). The amount of RIT delivered to tumor cells in quintile 5 following a 7.5 mg/kg dose is 1.7-fold higher than the amount per cell necessary to completely arrest growth, but 2-fold lower than the amount necessary to induce maximal cytotoxic activity, indicating some, but not maximal cytotoxic activity following that treatment. The amount of RG7787 delivered to quintile 5 following a 2.5 mg/kg dose is 7.2-fold lower than the amount necessary to induce maximal cell killing, indicating insufficient drug delivery to induce significant tumor regression due to cytotoxicity following treatment at that dose.

## Discussion

To better understand anti-tumor responses of immunotoxins and other immunoconjugates, we developed a general method to quantify the number of cytotoxic molecules internalized per cell in excised solid tumors and show a functional correlation between the amount of immunotoxin internalized *in vivo* and *in vitro* dose equivalents. We used the method to measure the internalization of RG7787 in KLM-1 tumors growing in mice, and found that following treatment with 7.5 mg/kg, sufficient molecules of RG7787 are delivered per cell to kill cells that have taken up the most RG7787 and to cause growth inhibition or arrest in many other cells. This was accompanied by the tumor regressions. This finding guides further improvements of immunotoxin treatment of mesothelin-expressing cancers, and this method will be used to quantify efficacy of such developments. Additionally, this method could be expanded to measure delivery of any biological agents that rely on internalization for function.

One useful feature of this approach is that it is designed to measure drug delivery in a variety of solid tumor xenograft models in mice because we used the widely and highly expressed human transferrin receptor (CD71) to identify human cancer cells^20^,^21^,^22^,^23^,^24^,^25^. Distinguishing murine cells from human cells is important for fully understanding drug delivery in preclinical xenograft models. Because the toxin used in these studies has very few lysine residues, the fluorochrome is mainly conjugated to lysine residues located in the Fab fragment of the RIT, and does not measure the amount of toxin fragment that has reached the cytosol, where it is able to block protein synthesis and induce apoptosis. Nevertheless, focusing on internalized RIT eliminates measurement of RIT molecules that do not contribute to efficacy, like those bound to the surface of tumor cells but not internalized, those internalized by stromal cells, and those remaining in the extracellular fluid, that would be measured by microscopy methods or methods lacking the ability to identify tumors cells^19^. This method is designed to measure uptake of any protein based therapeutic that relies on internalization for activity such as antibody-drug conjugates or protein-containing nanoparticles^27^,^28^.

Because of the wide range of cellular uptake revealed by the fluorescence-based method, we decided to partition the data into quintiles to better correlate uptake and response. Because of tumor heterogeneity and barriers to drug uptake, we were not surprised by the wide range of uptake^3^,^4^ we observed in this study. Partitioning was very useful and allowed us to directly compare the amount of RIT delivered to the amount necessary to kill cells. By using an absolute quantitative measure, like MESF, we could directly relate *in vitro* and *in vivo* model systems taking advantage of the fact that *in vitro* cells can be studied more easily. By extrapolating from how much RIT is needed to kill cells *in vitro*, we could determine whether sufficient RIT is delivered *in vivo* to tumor cells on average or to a portion of the tumor. Comparing *in vitro* and *in vivo* data like this can be used to quantify sufficiency of drug delivery, as demonstrated in this study, however it could also be used to give clear evidence of *in vivo* drug resistance. If tumors cells had internalized enough RIT to kill cells but there was no evidence of tumor regression, the data would be indicative of an *in vivo* resistance mechanism. We emphasize that measuring delivery of drug to tumor cells and determining whether enough drug is delivered, gives important information addressing why a treatment may not work, that can not be determined from methods that only measure relative drug delivery. We plan to use this flow cytometry method to investigate approaches to improve efficacy of RG7787 by using agents that may increase the amount of RIT internalized in individual tumor cells, increase the proportion of the tumor cells internalizing higher levels of RIT, and by sensitizing cells to RG7787 to decrease the number of RIT molecules necessary.

Approaches to increase internalization and tumor penetration could include altering the tumor microenvironment to increase perfusion using molecules like PEGPH20 that break down extracellular matrix components^29^,^30^, altering the affinity of the targeting domain^9^, or increasing half life in the circulation. While lower affinity monoclonal antibodies have been found to penetrate tumors more effectively than higher affinity antibodies^15^, it has also been shown that RIT bound to shed mesothelin can improve tumor penetration by acting as a reservoir^31^, suggesting that higher affinity to shed mesothelin would increase penetration.

Recently, we showed that both RG7787 and paclitaxel alone induced growth arrest in KLM-1 tumors, but when combined induced complete or near complete tumor regressions. Unlike in other models, the increased activity on KLM-1 tumors was not related to increased RIT internalization by tumor cells^13^,^19^. This indicates that combining RIT with chemotherapy drugs like paclitaxel may increase the sensitivity of KLM-1 tumor cells to killing by RG7787, and thereby decrease the number of RG7787 molecules that must be internalized. This method could be used to quantify this effect by measuring the number of molecules of RG7787 needed to kill cells in the presence of paclitaxel, in a similar manner as shown in Fig. 4A,B.

## Conclusions

We developed a general method to quantify the number of molecules of a therapeutic protein internalized into cells in solid tumors and correlated uptake *in vivo* with cell killing *in vitro*. Surprisingly we found no evidence that factors other than impaired drug entry contributed to the response of the tumors. Being able to quantify drug delivery at the cellular level and correlate it with expected activity should help in the design and development of novel therapeutic strategies.

## Materials and Methods

### RITs

Clinical grade RG7787 was manufactured by Roche Innovation Center, Penzberg, Germany. RG7787 is a re-engineered version of SS1P, shown in Fig. 1. Structural details of RG7787 and activity in PDAC were recently described^13^. RG7787 was labeled using the Alexa Fluor 647 Protein Labeling Kit (Invitrogen) for 3.5 hrs at room temperature, purified and quantified according to manufacturer’s instructions.

### Cell culture

PDAC cell line KLM-1 was provided by Dr. Udo Rudloff (National Cancer Institute (NCI), Bethesda, MD). Its identity was verified using short tandem repeat analysis in August 2012 (NCI, Frederick, MD). HCC70 triple negative breast cancer cell line was obtained from ATCC in October 2012. A431/H9 is a mesothelin-transfected epidermoid carcinoma cell line verified by short tandem repeat analysis in August 2014. All cell lines were maintained in RPMI-1640 supplemented with 10% FBS, 2 mmol/L L-glutamine, 1mmol/L sodium pyruvate, 100 U/mL penicillin and 100 μg/mL streptomycin (Invitrogen). Cells were maintained at 37 °C in a humidified incubator, with 5% CO_2_.

### Mouse experiments

Xenografts were grown subcutaneously in female athymic nude mice (NCr-Nu/Nu 01B74; NCI, Frederick, MD). KLM-1 tumors were established as described^13^. 2 × 10^6^ HCC70 or A431/H9 cells in 200 μl of RPMI-1640 with 4 mg/ml of Matrigel were injected into the intramammary fat pad or subcutaneously on the flank, respectively. We measured tumor length and width by electronic calipers and calculated volume using the formula (length × width^2^) ×0.4. Treatment began when tumors reached 120-140 mm^13^. RG7787, RG7787-Alexa Fluor 488 or RG7787-Alexa Fluor 647 was injected intravenously in 200 μl D-PBS 0.2% human serum albumin (HSA). Animals were handled according to NIH guidelines; the animal protocol was approved by the NCI Animal Care and Use Committee.

### *In vivo* RG7787 internalization in xenografts

Animals were treated with RG7787-Alexa Fluor 647 or 488 when tumors reached an average of 120-140 mm^13^. Animals were sacrificed at prescribed times, and tumors were excised. Digested tumor samples were prepared as previously reported^13^. Samples were stained with 10 μg/mL rat anti-mouse CD16/32 (BD Pharmingen) in D-PBS 5% FBS 1 mM EDTA. Samples were stained with anti-CD71 R-PE (transferrin receptor; BioLegend) or anti-EGFR R-PE (BD Pharmingen) followed by staining with SytoxBlue Dead Cell Stain (Life Sciences). Data were collected using an LSRII (BD Biosciences). Gating and data analysis were done in FlowJo 10 software (Tree Star, Inc.). Cells were gated to exclude SytoxBlue stained cells and doublets, tumor cells were selected by gating for CD71 or EGFR positive cells (gating for representative control sample in Supporting Information Fig. 4). The amount of internalized RG7787-Alexa Fluor 647 was assessed by measuring the MFI of Alexa Fluor 647 in the tumor cell populations.

### Quantification of internalized RG7787 molecules

Alexa Fluor 647 Quantum MESF (Bangs Laboratories) were run before test samples. MESF beads were used to create a standard curve comparing MESF and MFI. Using the QuickCal Analysis Template v. 2.3 (Bangs Laboratories), the MFI of *in vivo* or *in vitro* test samples was interpolated to find MESF. The number of equivalent soluble Alexa Fluor 647 molecules derived from QuickCal was converted into the number of RG7787-Alexa Fluor 647 molecules by first subtracting auto-fluorescence based on control samples and then correcting for number of Alexa Fluor647 molecules per RIT by using the labeling efficiency measured in the Alexa Fluor 647 Protein Labeling kit.

### *In vitro* immunotoxin internalization

KLM-1 cells (2 × 10^5^) were seeded in 6-well dishes. After 16-20 hrs, cells were treated with different RG7787-Alexa Fluor 647 concentrations in 0.6 mL of media for 24 hrs. Surface bound RG7787-Alexa Fluor 647 was stripped by using a low pH glycine buffer (0.2 mol/L glycine-HCl, pH 2.5). Cells were harvested from plates and analyzed by flow cytometry.

### *In vitro* activity

Cell death was measured by seeding 2 × 10^5^ KLM-1 cells in 6-well dishes and treating with various doses of RG7787 in 0.6 mL of media after 16-20 hrs. At the time of treatment one well was counted to quantify the starting number of cells. After 24 hrs, wells were washed with D-PBS, and RG7787 was replaced with media; 48 hrs later, cells were counted in triplicate on a Cellometer Vision (Nexcelcom) using Trypan blue staining to exclude dead cells.

### Statistics

Data points were collected independently at least in triplicate with data shown as mean ± standard error of measurement (SEM) or representative values. *In vivo* efficacy assessment was repeated and representative experiments are shown. Tumor volumes are compared using Student’s t-tests. Analysis of SEM and statistical significance was done using GraphPad Prism 6 (GraphPad Software, Inc.). P-values of less than 0.05 were considered significant.

## Additional Information

**How to cite this article**: Mason-Osann, E. *et al*. Quantification of recombinant immunotoxin delivery to solid tumors allows for direct comparison of *in vivo* and *in vitro* results. *Sci. Rep*. **5**, 10832; doi: 10.1038/srep10832 (2015).

## Supplementary Material

Supplementary Information
