# Supplementary material for: Quantification of recombinant immunotoxin delivery to solid tumors allows for direct comparison of in vivo and in vitro results
Source: Sci Rep. 2015 Jun 26;5:10832. doi: 10.1038/srep10832 (PMC4482048; doi:10.1038/srep10832)

Quantification of recombinant immunotoxin delivery to solid tumors allows for direct comparison of *in vivo* and *in vitro* results

Emily Mason-Osann[1,2], Kevin Hollevoet[1,3], Gerhard Niederfellner[4], & Ira Pastan[1]

[1]Laboratory of Molecular Biology, Center for Cancer Research, National Cancer Institute, National Institutes of Health, Bethesda, Maryland, USA

[4]Roche Pharma Research & Early Development, Roche Diagnostics GmbH, Nonnenwald 2, 82377 Penzberg, Germany

SUPPORTING INFORMATION

Supporting Information Figure Legends

Figure S1. Identification of human tumor cells in different xenograft models. Data shown is KLM-1 (A-B), HCC70 (C) or A431/H9 (D) control tumors that were excised, digested, stained, and single live cells were selected. In KLM-1 tumor that was stained with anti-human EGFR R-PE (A) and anti-mouse F4/80 human tumor cells (Q3) are not clearly distinguishable from murine unstained murine cells (Q4) unlike in KLM-1 tumors stained with anti-human CD71 R-PE (B). C-D. HCC70 (C) or A431/H9 (D) tumor stained with anti-human CD71 R-PE. Human tumor cell populations (Q3) show distinguishable separation from murine cells (Q4).

Figure S2. Quantification of RG7787 molecules per cell using Alexa Fluor 647 MESF beads as a standard. Alexa Fluor 647 MESF beads were run at the same time as test samples using the same instrument settings. MFI of each bead population was measured (A) and plotted using QuickCal software from Bangs Laboratories (B). This standard curve ($R \geq 0.9953$) was used to interpolate MESF values of test samples, which were converted to RG7787 molecules per cell using the labeling efficiency of RG7787-Alexa Fluor 647.

Figure S3. *In vitro* internalization of saturating dose of RG7787-Alexa Fluor 647 over time. KLM-1 cells were plated ($2x10^5$) for 16-20 hrs and then treated with 2μg/ml RG7787-Alexa Fluor 647 in 0.6mL for given amount of time before

being surface stripped, harvested and analyzed by flow cytometry. Data shown is average of triplicate experiments with SEM.

Figure S4. Flow cytometry-gating strategy to identify tumor cell population. Data shown in a control (D-PBS 0.2% HSA treated) KLM-1 tumor that has been excised, digested, and stained with anti-human CD71 R-PE and Sytox Blue. A. Exclude cells that stain for Sytox Blue and debris to identify live cell population. B-C. Exclude doublets using forward scatter (FSC) and side scatter (SSC) signal width vs. height. D. Identify human tumor cells from murine cells in the xenograft by selecting cells positive for anti-CD71 R-PE stain. Use tumor cell population for further analysis.

Supporting Information Fig. S1

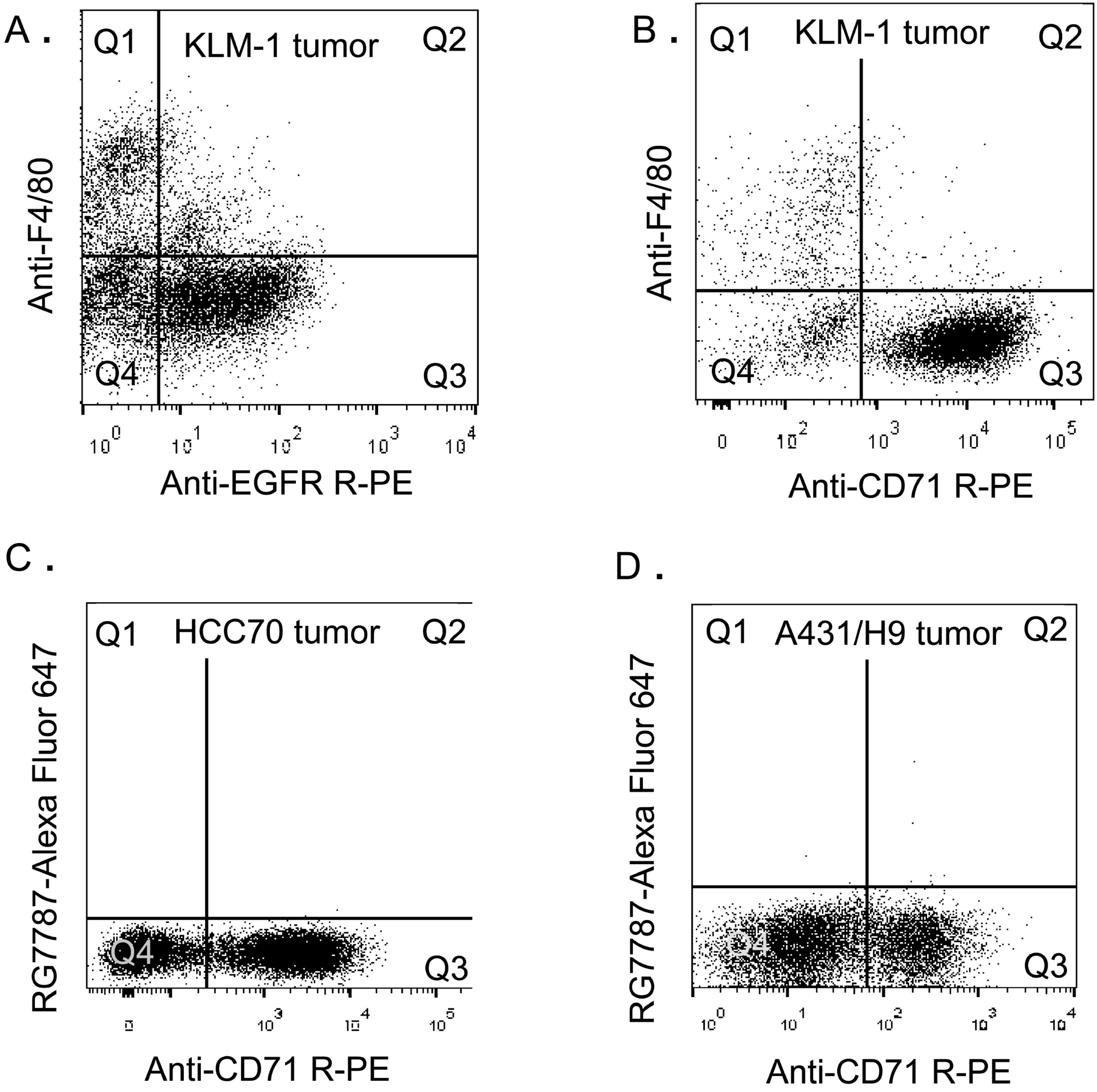

Supporting Information Fig. S2

A.

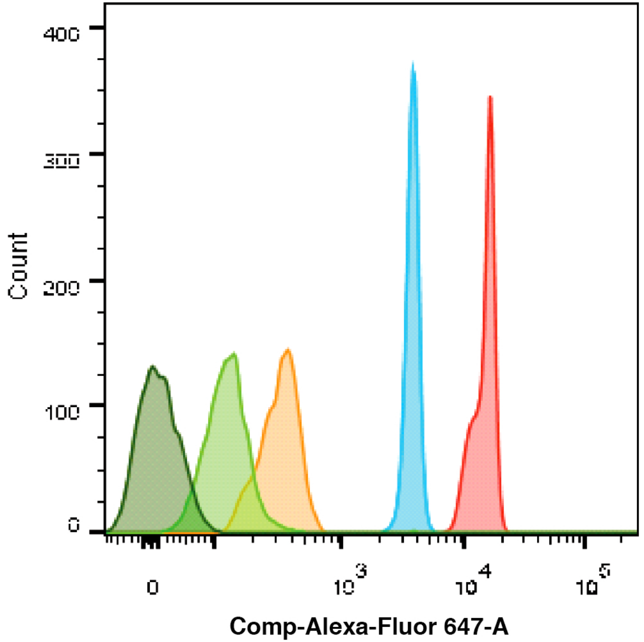

	TUBE NAME	Geometric Mean : Comp-Alexa Fluor 647-A
	Blank bead	9.58
	Bead 1	134
	Bead 2	321
	Bead 3	3629
	Bead 4	14110

B.

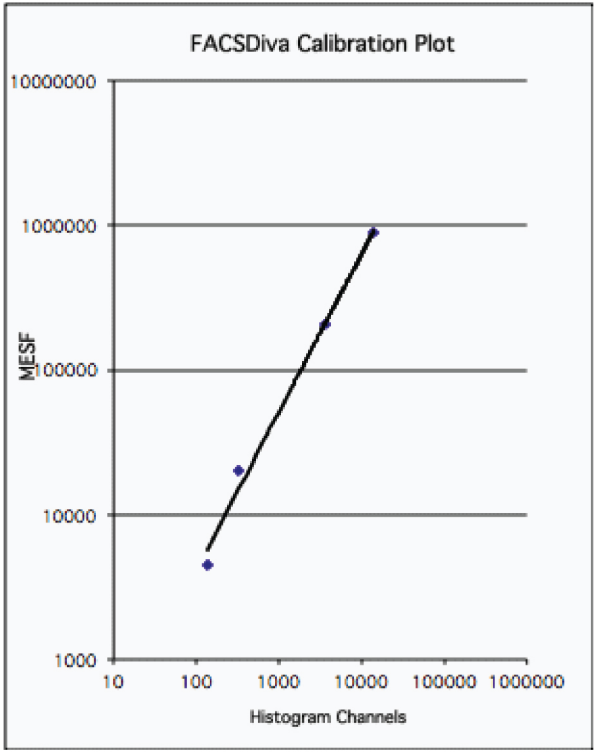

Supporting Information Fig. S3

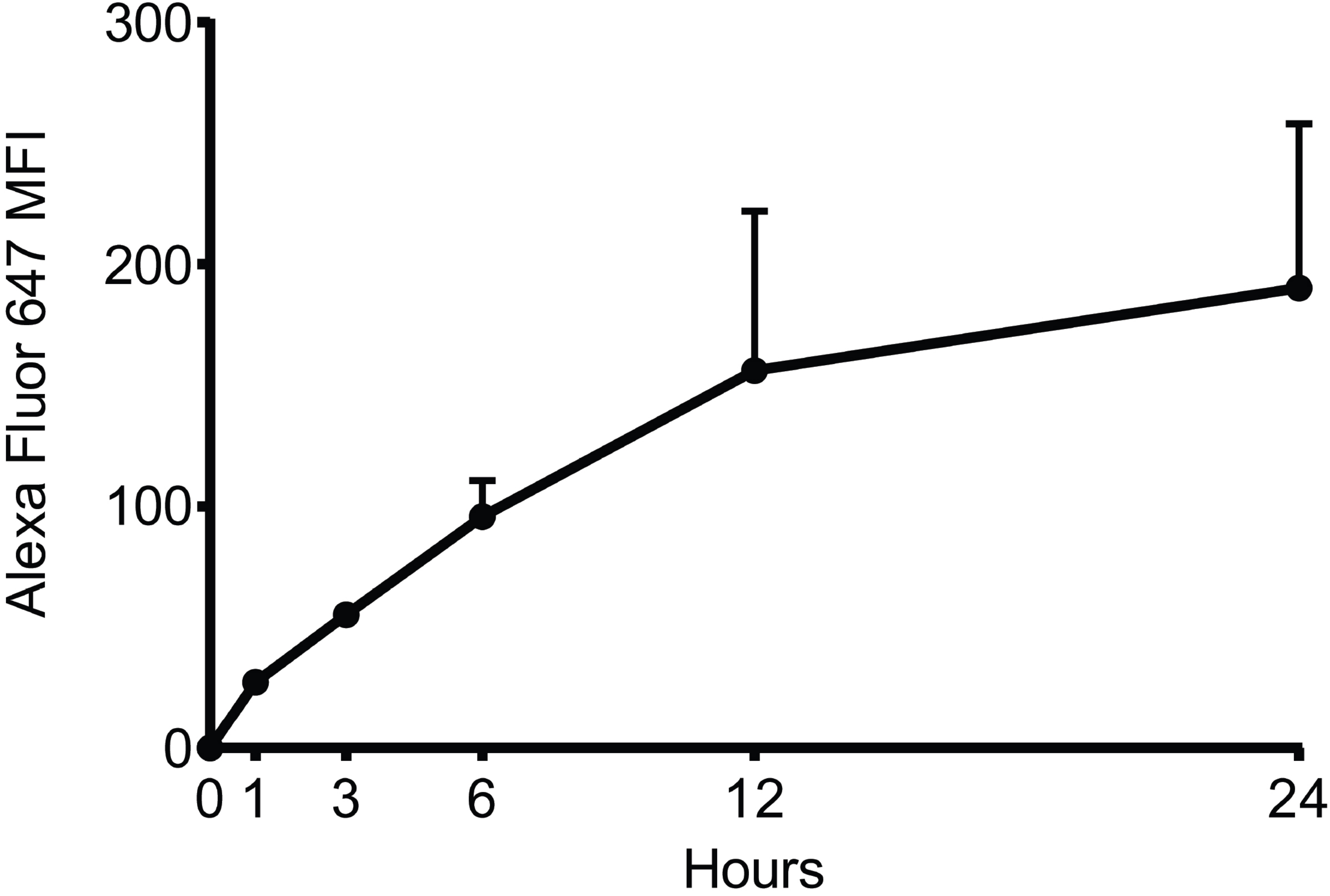

Supporting Information Fig. S4

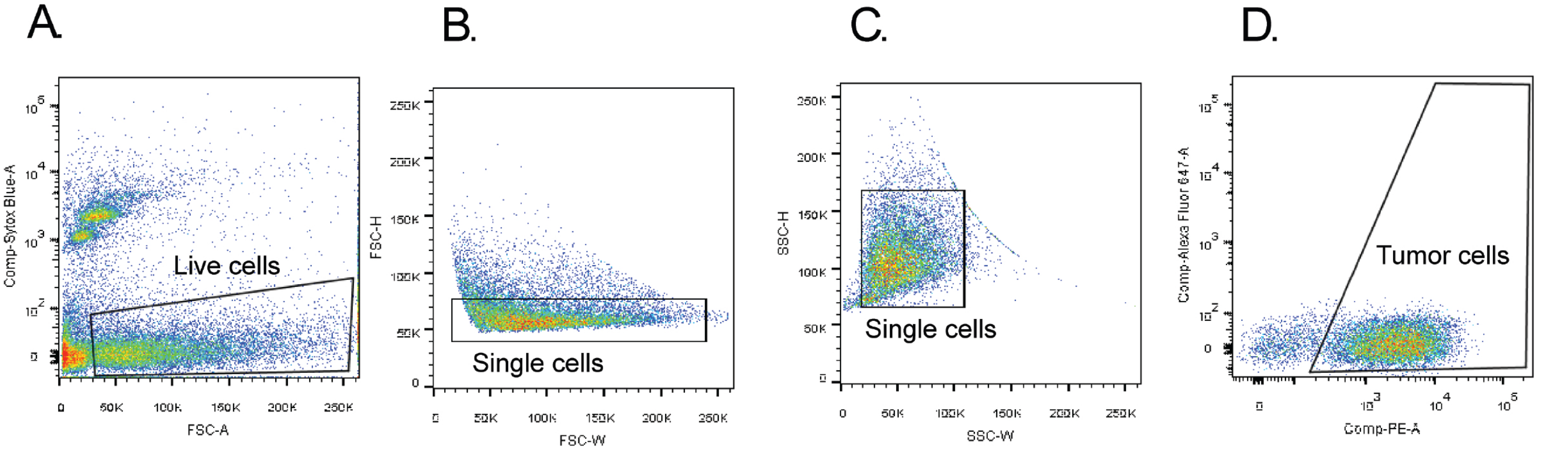

Supplement: Supplementary Information [file srep10832-s1.pdf]
